# Enriched retinal ganglion cells derived from human embryonic stem cells

**DOI:** 10.1038/srep30552

**Published:** 2016-08-10

**Authors:** Katherine P. Gill, Sandy S. C. Hung, Alexei Sharov, Camden Y. Lo, Karina Needham, Grace E. Lidgerwood, Stacey Jackson, Duncan E. Crombie, Bryony A. Nayagam, Anthony L. Cook, Alex W. Hewitt, Alice Pébay, Raymond C. B. Wong

**Affiliations:** 1 Centre for Eye Research Australia, Royal Victorian Eye and Ear Hospital & Ophthalmology, Department of Surgery, the University of Melbourne, Australia; 2 National Institute of Aging, National Institutes for Health, USA; 3 Monash Micro Imaging, Monash University, Australia; 4 Department of Otolaryngology & Department of Surgery, the University of Melbourne, and Royal Victorian Eye and Ear Hospital, Australia; 5 Departments of Audiology and Speech Pathology & Ophthalmology, University of Melbourne, Australia; 6 Wicking Dementia Research and Education Centre, University of Tasmania, Australia; 7 School of Medicine, Menzies Institute for Medical Research, University of Tasmania, Australia

## Abstract

Optic neuropathies are characterised by a loss of retinal ganglion cells (RGCs) that lead to vision impairment. Development of cell therapy requires a better understanding of the signals that direct stem cells into RGCs. Human embryonic stem cells (hESCs) represent an unlimited cellular source for generation of human RGCs *in vitro*. In this study, we present a 45-day protocol that utilises magnetic activated cell sorting to generate enriched population of RGCs via stepwise retinal differentiation using hESCs. We performed an extensive characterization of these stem cell-derived RGCs by examining the gene and protein expressions of a panel of neural/RGC markers. Furthermore, whole transcriptome analysis demonstrated similarity of the hESC-derived RGCs to human adult RGCs. The enriched hESC-RGCs possess long axons, functional electrophysiological profiles and axonal transport of mitochondria, suggestive of maturity. In summary, this RGC differentiation protocol can generate an enriched population of functional RGCs from hESCs, allowing future studies on disease modeling of optic neuropathies and development of cell therapies.

Optic neuropathies represent a group of ocular diseases characterized by the loss of retinal ganglion cells (RGCs), including glaucoma, Leber’s Hereditary Optic Neuropathy (LHON), and autosomal dominant optic atrophy (ADOA). Glaucoma is one of the leading causes of irreversible blindness in the developed world^1^ and is characterised by progressive RGC death and subsequent vision loss^2^. Unlike glaucoma, LHON is a maternally inherited disease^3^. LHON is a mitochondrial genetic disease classified by acute or subacute vision loss, predominantly affecting young males^4^,^5^. LHON is caused by mitochondrial mutations in Complex I subunits, leading to defects in bioenergetics output and ultimately RGC apoptosis in patients^6^. On the other hand, ADOA progresses insidiously from a young age, presenting with symmetric loss of central vision due to death of RGCs and loss of myelin in the optic nerve^7^. Together, these optic neuropathies represent a large proportion of people affected by vision loss. However, a major barrier to the study of optic neuropathies is the difficulty in obtaining RGC samples from patients pre-mortem. Currently there is no mammalian RGC line available for the study of optic neuropathies. Many previous studies have relied on the cell line RGC-5, previously thought to be a transformed rat RGC line^8^. However, recent re-characterization identified the RGC-5 line as a mouse photoreceptor cell line^9^,^10^. Thus, generation of an *in vitro* RGC model is crucial to advance research on optic neuropathies.

Human pluripotent stem cells represent a renewable cellular source for generating RGCs. In particular, human embryonic stem cells (hESCs) and human induced pluripotent stem cells (hiPSCs) are capable of indefinite proliferation and can differentiate into all cell types of the body^11^,^12^,^13^,^14^. Patient-specific hiPSCs can be used for disease modeling to study the pathophysiology of optic neuropathies *in vitro*, such as glaucoma^15^,^16^. Additionally, studies using hiPSCs for modeling other retinal diseases have gained momentum in recent years, as evidenced by studies on Best disease^17^ and retinitis pigmentosa^18^,^19^. Several research groups have established that hESCs and hiPSCs can be differentiated into multiple retinal cell types, including RGCs, retinal pigment epithelium (RPE) cells, and photoreceptors^20^,^21^. However, many current differentiation protocols yield a heterogeneous population of retinal cells, containing only a low proportion of RGCs^22^. This remains an issue for using hiPSCs for modeling optic neuropathies and development of RGC replacement therapy.

In the present study, we report the development of an improved protocol for differentiation and enrichment of RGC cultures from hESCs. With this method we demonstrate that hESCs can be differentiated into retinal progenitor cells (RPCs) and subsequently RGCs. Using magnetic activated cell sorting (MACS), we isolated RGCs from the heterogeneous retinal differentiated culture. Following cell sorting, we showed that the enriched RGC population is functional, as demonstrated by electrophysiological recordings and mitochondrial axonal transport. This study provides a protocol for generating an enriched population of RGCs using hESCs which can be used for modeling of optic neuropathies and RGC replacement therapy.

## Results

### Step-wise retinal differentiation to direct hESCs to RPCs and RGCs

**Figure 1:**
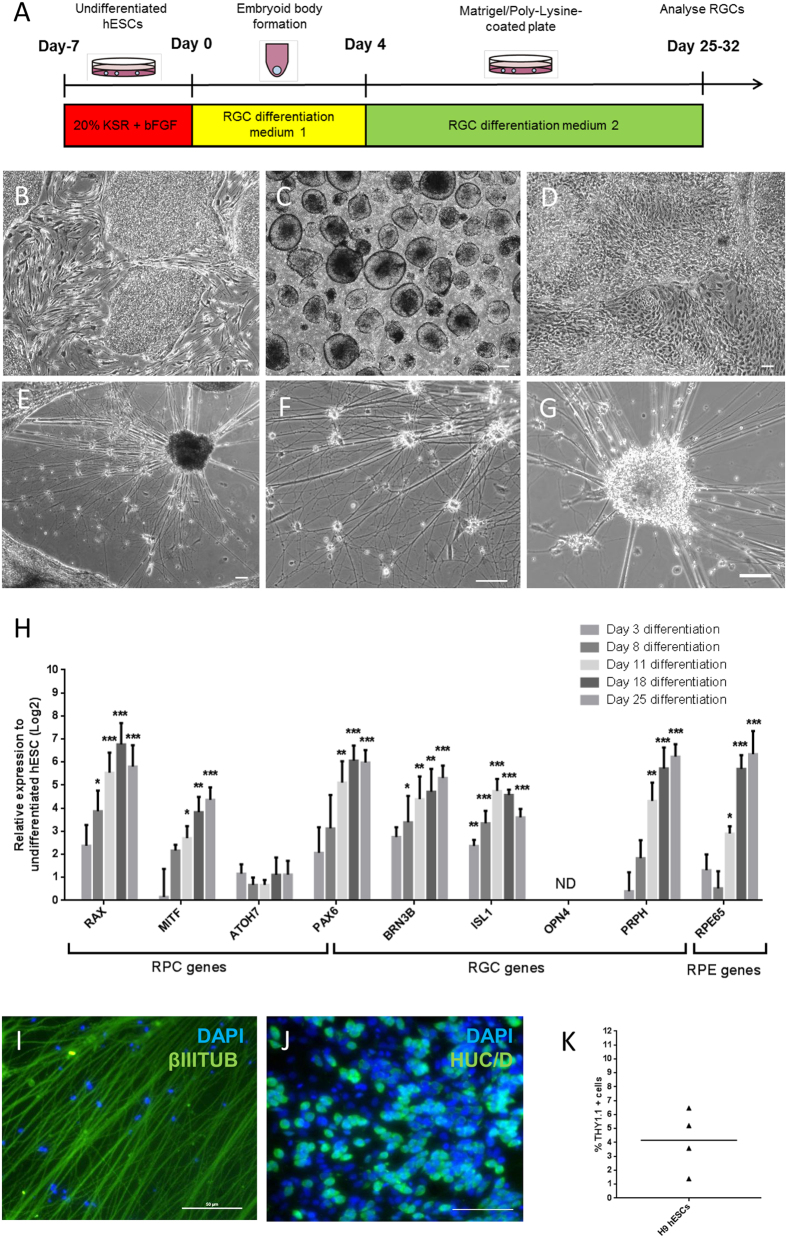
Retinal differentiation of hESCs. (**A**) Schematic timeline of retinal differentiation using hESCs. (**B–G**) Phase contrast images showing cell morphology at different time points during retinal differentiation: (**B**) day 0 undifferentiated hESCs, (**C**) day 3 embryoid bodies, (**D**) day 10, (**E**) day 21 and (**F**) day 27 culture with extensive neuronal network and (**G**) clusters of neurons. Scale bars = 100 μm. (**H**) qPCR analysis of gene expression associated with RPCs (*ATOH7, RAX, MITF*), RGCs (*BRN3B, ISL1, OPN4, PRPH*) and both RPCs/RGCs (*PAX6)* and RPE (*RPE65)* during retinal differentiation at different time points, n = 3–4, error bars represent SEM, *p < 0.05, **p < 0.01, ***p < 0.001. ND: not detected. Immunocytochemistry analysis of day 25 hESC-derived RGCs with (**I**) βIII TUBULIN or (**J**) HU C/D with the corresponding DAPI. Scale bars for (I) 50 μm; (**J**) 100 μm. (**K**) Quantification of THY1.1 positive cells at day 30 post-retinal differentiation in hESCs (n = 4).

Previous work demonstrated the feasibility of deriving RGCs from hESCs^21^. We modified this protocol to extend the duration of embryoid body formation and utilise 10% knockout serum replacement for retinal differentiation (Fig. 1A). Using this modified protocol, we directed hESCs into retinal differentiation by activation of Insulin-like Growth Factor (IGF) signaling using IGF1, together with inhibition of Wnt signaling and Bone Morphogenetic Protein (BMP) signaling using DKK1 and Noggin, respectively (Fig. 1A). Undifferentiated H9 cells were allowed to form embryoid bodies for four days and plated down for further differentiation (Fig. 1B,C). The differentiated culture was highly heterogeneous after 10 days (Fig. 1D). Differentiated cells with neuronal morphology were observed after 21 days of differentiation (Fig. 1E). By day 27, extensive networks of neurons (Fig. 1F) and clusters of neurons (Fig. 1G) were observed.

To determine the efficacy of hESCs to differentiate into the retinal/RGC lineage, we performed qPCR to examine gene markers for RPCs, RPE cells and RGCs (Fig. 1H). Our qPCR analysis indicated a gradual increase of *MITF* expression from day eight onwards, which marks the RPCs at the optic vesicle stage as well as the RPE cells^23^. Expression of another marker, *ATOH7*, which is required for RGC specification^24^,^25^,^26^, showed a modest increase from day three onwards. The expression of the RPC marker *RAX* peaked at day 18^27^. By day 25, expression of the eye field transcription factor *PAX6* was significantly upregulated, which is an important regulator for RGC development and its expression persist in RGCs^28^. Importantly, strong expression of *BRN3B* and *ISL1* were detected in the day 25 cultures, both are RGC markers within the retina^29^,^30^. However, *OPN4* expression was not detected at any stage during retinal differentiation, suggesting the absence of a small subtype of intrinsically photosensitive RGCs^31^. *PRPH*, a marker for peripheral neurons and RGCs^32^,^33^, was significantly upregulated from day 11 onwards. Finally, *RPE65* expression was also upregulated and peaked at day 25, indicating the presence of RPE cells within the differentiated culture. Together, these results suggest this step-wise differentiation protocol directed hESCs to differentiate along the retinal lineages into RPCs, RPE cells and RGCs.

To further characterize the hESC-derived RGCs, immunocytochemistry was performed with the day 25 cultures. Within the population, hESC-derived RGCs were observed with positive expression of the neuronal marker βIII TUBULIN (Fig. 1I) and RGC/amacrine cell marker HU C/D (Fig. 1J). Patch-clamp electrophysiology determined functionality of the hESC-derived RGCs with the ability to fire action potentials (data not shown). Together, these results suggest successful differentiation of hESCs into retinal neurons using this modified retinal differentiation protocol.

### Enrichment of hESC-derived RGCs following step-wise retinal differentiation

To measure the efficiency of RGC differentiation by this protocol, we quantified the percentage of hESC-derived RGCs in the culture after 30 days of retinal differentiation. Our results showed that only 4.2 ± 1.1% (n = 4) of RGCs were present within the differentiated culture, using THY1.1 as a RGC marker within the retina (Fig. 1K). We also observed a similar RGC differentiation efficiency at ~4% using hiPSCs (data not shown).

**Figure 2:**
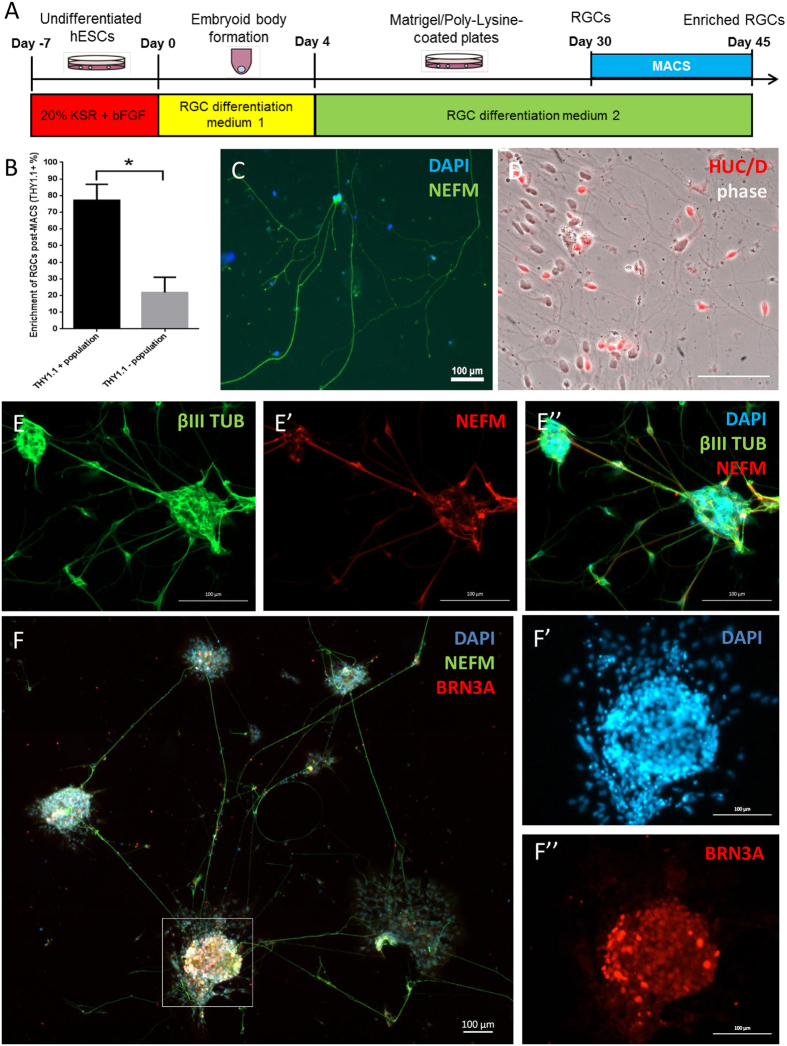
Enriched hESC-derived RGCs by MACS. (**A**) Schematic timeline of procedure to isolate hESC-derived RGCs by MACS. (**B**) Quantification of THY1.1 positive RGCs post-MACs. (**C–F**) Characterisation of enriched hESC-derived RGCs using a panel of known RGC markers: (**C**) NEFM; (**D**) HUC/D; (**E**) E’ and E”) individual and merged images of NEFM and βIII TUBULIN; (**F**) co-staining of BRN3A and NEFM. The magnified images of the highlighted region are shown in F’) as DAPI and F”) as BRN3A. n > 3 for all markers. Scale bars = 100 μm.

As this RGC differentiation protocol is inefficient and yields a heterogeneous population of cells, it is desirable to purify the hESC-derived RGCs for subsequent biochemical/cellular analysis. THY1.1 is a surface marker previously used for derivation of primary RGCs in rat, mouse and human^34^,^35^,^36^. We thus tested the feasibility of utilising MACS to enrich for THY1.1 positive RGCs. We performed MACS enrichment for THY1.1 positive RGCs on day 30 and the enriched cells were re-plated to allow for another 15 days of differentiation prior to analysis (Fig. 2A). Once replated, we observed that the cells would grow sparse as well as in clusters, as shown in Fig. 2C–F. Using flow cytometry analysis we quantified the enrichment of RGCs following MACS isolation. As shown in Fig. 2B, our results indicated that MACS enrichment yielded 77.2 ± 9.6% THY1.1 + cells on day 30, compared to a statistically significant lower percentage of RGCs in the THY1.1 negative population (21.9 ± 9.1% THY1.1+ cells), suggesting successful RGC enrichment using MACS. By day 45, we observed an extensive neuronal network of hESC-derived RGCs, containing cluster of cells and dissociated cells, with very long neurites that are typical of RGCs (Fig. 2C,D). Enriched hESC-RGCs were characterised by immunocytochemistry using a panel of five RGC-associated markers. We detected nuclear expression of HU C/D (Fig. 2F) and BRN3A (Fig. 2E), the latter being an important transcription factor for RGC specification^37^, as well as cytoskeletal expression of Neurofilament M (NEFM, Fig. 2C) and βIII TUBULIN (Fig. 2D). Also, strong expression of THY1 remained in the hESC-derived RGCs following prolonged culture (Suppl. Fig. 1). On the other hand, we did not detect expression of CRALBP and RPE65 by immunocytochemistry, confirming the absence of Müller cells and RPE cells respectively (Suppl. Fig. 2).

### Transcriptome analysis reveals similarity of enriched hESC-RGCs to RGCs *in vivo*

**Figure 3:**
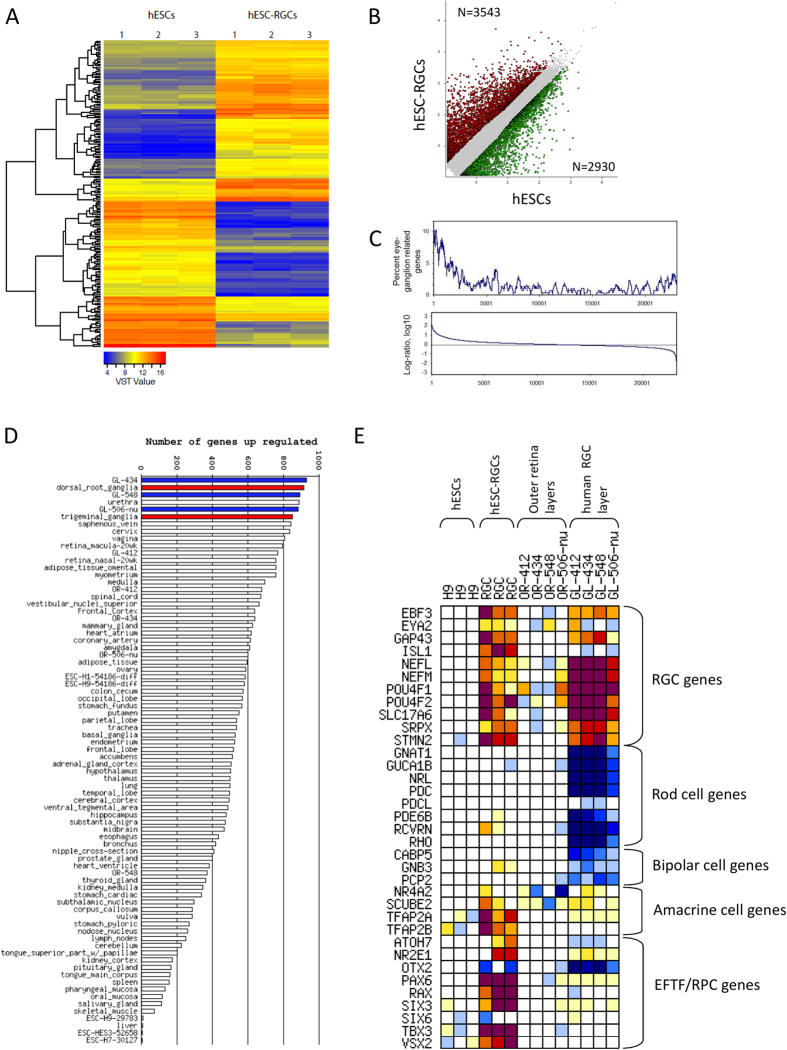
Transcriptome profiling shows similarity in enriched hESC-derived RGCs to *in vivo* RGCs. (**A**) Heatmap displaying the top 200 differentially expressed genes in undifferentiated hESCs and enriched hESC-RGCs following Variance Stabilizing Transformation (VST). (**B**) Scatter-plot comparing expression profiles of undifferentiated hESCs and enriched hESC-RGCs. Red spots represent upregulated genes and green spots represent downregulated genes in hESC-RGCs compared to undifferentiated hESCs, with statistical significance of FDR < 0.05 and > 2 fold changes. (**C**) Rank plots show enrichment of retinal ganglion related genes^33^ among genes upregulated in hESC-RGCs. (**D**) Rank-plot based on number of upregulated genes in hESC-RGCs that match to upregulated genes in various human tissues, with expected proportion of false positives^70^ (EPFP = 0.3). Upregulated genes are normalised to undifferentiated H9 hESCs. Blue bars represent human RGC layer samples^33^ and red bars represent the sensory neurons. (**E**) Heatmap displaying gene expression of markers of RGCs, rod cells, bipolar cells, amacrine cells and eye field transcription factor (EFTF)/ RPCs. hESC-RGCs expression is displayed as log-ratio change relative to hESCs. Human RGCs expression is displayed as log-ratio change relative to the outer retina layers^33^.

To further characterize and confirm the identity of the enriched hESC-RGCs, we performed whole transcriptome analysis using RNA sequencing (seq). Figure 3A shows a heatmap of the top 200 differentially expressed genes in undifferentiated hESCs and enriched hESC-RGCs (Day 45). Our analysis detected 3,543 upregulated genes and 2,930 downregulated genes in hESC-RGCs compared to hESCs (Fig. 3B, >2 fold changes with FDR < 0.05). We compared the transcriptome profile of hESC-RGCs to adult human RGCs previously isolated by laser capture microdissection^33^. Gene set enrichment analysis revealed an overall enrichment of RGC-related genes in the hESC-RGCs, supporting the RGC identity of the hESC-RGCs (Fig. 3C). Next, we expanded the comparison of transcriptome profile of hESC-RGCs to 78 various human tissues/cell types obtained from the NCBI GEO database. As shown in Fig. 3D, out of the top 6 ranked tissues based on transcriptome similarities, 3 of them were adult RGC samples (ranked 1^st^, 3^rd^ and 5^th^) and 2 of them were sensory ganglia (ranked 2^nd^: dorsal root ganglia, 6^th^: trigeminal ganglia), which are known to have similarities with RGCs as shown by a previous gene expression profiling study^38^. Together, these results provide strong support of similarities between enriched hESC-RGCs and human RGCs *in vivo*.

To determine whether the enriched hESC-RGC culture contained other retinal neurons, we assessed the gene expression of markers of rod cells, bipolar cells, amacrine cells and RPCs. As shown in Fig. 3E, enriched hESC-RGCs express multiple RGC genes (*POU4F1/BRN3A, POU4F2/BRN3B, ISL1, NEFL, NEFM, SLC17A6/VGLUT2, EBF3, EYA2, GAP43, SRPX and STMN2*). In particular, Neurofilament L (*NEFL)* and *NEFM* are intermediate filament proteins expressed in mature neurons^39^, with the former also shown to be critical in maturation of regenerating myelinated axons^40^. Both NEF L and M are expressed in axons of RGCs *in vivo*^41^. However, we did not detect significant upregulation of expression of *NES* / NESTIN in enriched hESC-RGCs compared to undifferentiated hESCs (data not shown), which is one of the earliest intermediate filaments associated with neuronal development^42^. Also, *SLC17A6* encodes for the vesicular glutamate transporter VGLUT2, a glutamatergic neuronal marker predominantly expressed by RGCs within the rat retina^43^.

In comparison, we detected no obvious marker expression for rod cells or bipolar cells. Out of the four amacrine cell markers analysed, only *TFAP2A* was upregulated in hESC-RGCs in a statistically significant manner, although variable expression of *TFAP2B and SCUBE2* were detected in some samples of hESC-RGCs. This may indicates possible carryover of a small population of amacrine cells in our culture following MACS. Finally, the hESC-RGCs also show high expression of multiple RPC genes (*PAX6, RAX, SIX3, TBX2, VSX2)*. Thus, it is possible that some residual RPCs and/or immature RGCs are present in the enriched hESC-RGC culture.

### Enriched hESC-RGCs possess functional electrophysiological properties

**Figure 4:**
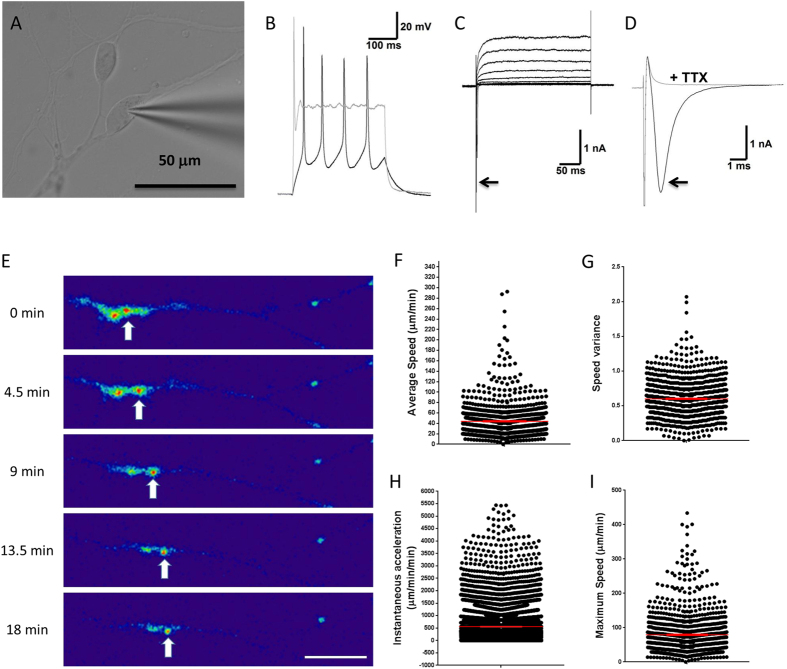
Electrophysiological profile and axonal transport of enriched hESC-derived RGCs. (**A**) RGCs with recording microelectrode attached. (**B**) Typical firing profile observed in response to membrane depolarisation: multiple action potentials are evident at threshold (black trace), whilst only a single action potential is observed at higher stimulation levels (grey trace). (**C**) Voltage-clamp recording shows activation of inward sodium currents (arrow) followed by outward potassium currents in response to increasing membrane depolarisation. (**D**) The inward sodium current (arrow; black trace) is abolished in the presence of 1 mM tetrodotoxin (TTX; grey trace), a potent blocker of voltage-gated sodium channels. (**E**) Time-lapse images showing axonal transport of mitochondria cluster (white arrows). Scale bar = 15 μm. Quantification of (**F**) average speed (n = 1,049), (**G**) variance in speed (n = 1,049), (**H**) instantaneous acceleration (n = 10,606) and (**I**) maximum speed of mitochondria transport along axon (n = 1,049). Mean ± SEM are shown in red.

We performed electrophysiological analysis to measure the function of enriched hESC-RGCs (Fig. 4A). Enriched hESC-RGCs were capable of firing multiple action potentials in response to membrane depolarisation (Fig. 4B). Voltage-clamp recording showed activation of a fast, transient inward current followed by a sustained outward current in response to increasing membrane depolarisation (Fig. 4C). In addition, the fast inward current was abolished by treatment with tetrodotoxin (TTX), confirming its mediation by sodium channels (Fig. 4D). Together, our results confirm that the enriched hESC-RGCs are functional and display an electrophysiological profile consistent with relatively mature neurons.

### Enriched hESC-RGCs are capable of axonal transport of mitochondria

Another characteristic feature of mature RGCs is their ability for axonal transportation of mitochondria to maintain homeostasis^44^. We performed time-lapse video imaging and measured the kinetics of axonal transport of mitochondria in enriched hESC-RGCs (Fig. 4E). Clusters of mitochondria were observed to move along axons (Fig. 4E). By tracking over 1,000 mitochondria clusters, we observed axonal transport of mitochondria in hESC-RGCs with an average speed of 44.01 ± 0.93 μm/min (Fig. 4F), reaching a maximum speed of 79.4 ± 1.8 μm/min (Fig. 4H) and an average instantaneous acceleration of 555.8 ± 6.1 μm/min/min (Fig. 4I). The transport of mitochondria clusters were observed to pause frequently, with cluster speed variance of 0.6 ± 0.008 (Fig. 4G). As indicated by the wide range of the maximum speed measured, both fast and slow mitochondrial transport was observed. We also observed bi-directional axonal transport of mitochondria in enriched hESC-RGCs.

## Discussion

Previous studies have reported stepwise differentiation of pluripotent stem cells by recapitulating the signaling pathways that occur during RGC development. Multiple signaling pathways are known to regulate retinogenesis, including those modulated by bFGF^45^,^46^,^47^,^48^, IGF-1^49^, sonic hedgehog (SHH)^47^ activin^50^,^51^, BMP^46^,^48^, Wnt^52^, Nodal^53^ and Notch^54^. Based on manipulation of these signaling pathways, many retinal differentiation protocols for human pluripotent stem cells are reported to yield a heterogeneous population of RPE cells, photoreceptors and RGCs. However, the derived RGCs are often not well characterized and there is a lack of protocol focusing on deriving RGCs specifically.

Recently, Riazifar *et al*. reported a 40-day RGC differentiation protocol that yielded up to 30% RGCs. The iPSC-derived RGCs were carefully characterised using six RGC markers and possessed functional electrophysiology^55^. Similarly, Tanaka *et al*. reported generation of iPSC-derived RGCs by formation of self-organising optic vesicles. By plating down these optic vesicles, the authors reported 90% of the optic vesicles yield RGC outgrowth that express multiple RGC markers, possess axonal transport and action potentials^56^. It is important to note that many markers used to identify RGCs are also expressed in other cell types. For examples, the BRN3 family of proteins is also expressed in auditory and somatosensory neurons^57^, while HUC/D is expressed in enteric neurons^58^. Therefore, identification of RGCs should be performed using multiple RGC markers coupled with functional analysis. In this regard, our study describes a protocol to yield enriched RGCs using hESCs. Here we demonstrated that hESCs can be differentiated towards the retinal lineage, as shown by up-regulation of retinal progenitor markers, and subsequently enriched to yield RGCs. hESC-derived RGCs generated by our protocol have extended long neurites. Transcriptome and immunocytochemistry analysis demonstrated that the enriched RGCs expressed a panel of gene and protein markers characteristic of RGCs, including *BRN3B, ISL1, PAX6, SLC177A6/VGLUT2, EBF3, EYA2, GAP43, SRPX, STMN2,* BRN3A, THY1, NEFM, HUC/D and βIII TUBULIN. Comparison with whole transcriptome provided strong support of similarities between hESC-derived RGCs and RGCs *in vivo.* Our results also highlighted the transcriptome similarities between human RGCs and sensory ganglia, an observation consistent with those reported in rats^38^. Therefore given the known similarities of RGCs and sensory neurons, we cannot exclude the presence of other sensory neurons in our enriched population. Functional analysis of the hESC-derived RGCs demonstrated electrophysiological functionality indicative of mature neurons, as well as functional mitochondrial axonal transport. Our protocol utilizes specific growth factors and small molecules to direct embryoid bodies along the retinal lineage to yield RGCs by stepwise differentiation and a THY1.1 MACS sorting technique to enrich the population of RGCs. We showed that although the RGC differentiation protocol is relatively inefficient (~4%), subsequent MACS enrichment can yield RGCs at a sizable scale for downstream cellular and biochemical assays. Future modifications of growth factor cocktails to promote retinogenesis will enhance RGC differentiation efficiency and further facilitate efficient generation of RGCs using human pluripotent stem cells. Another approach, although outside the scope of this study, could focus on obtaining higher number of retinal progenitors, which could potentially be achieved by replacing the embryoid body steps with a neurosphere or other neurally enriched organoid step.

Enrichment of RGCs from heterogeneous differentiated cultures is an important step for disease modeling and future cell-based therapies. Primary RGCs have been isolated from human and rodent samples by a range of methods, such as laser capture micro-dissection of the RGC layer^59^, or more commonly using the surface marker THY1.1 or THY1.2 for immunopanning or MACS isolation^32^,^38^,^49^,^60^,^61^,^62^. Despite this, many any current hESC/hiPSC retinal or RGC differentiation protocols do not incorporate a purification or enrichment step to isolate the RGC population. During the revision of this manuscript, Sluch *et al*. described the generation of a BRN3A reporter hESC line and subsequent purification of RGCs using FACS, providing a significant step to improve the purity of stem cell-derived RGCs^63^. Compared to a nuclear marker, the use of a surface marker to isolate RGCs will further improve the applicability of the RGC differentiation methodology. In this regard, a brief report by Tucker *et al*. utilized MACS to isolate THY1 positive RGC-like cells with neuronal morphology in hiPSCs^16^. Building on these advances, our protocol addresses the issues of heterogeneity by utilising MACS for isolation of THY1.1 positive RGCs and we have performed extensive marker analysis, transcriptome analysis and functional assays to confirm their identity. However, our results indicate that residual non-RGCs, possibly amacrine cells or RPCs, can remain in culture following MACS enrichment. This should be taken into consideration when interpreting the presented results and designing downstream assays.

The recent development of three dimensional-organoid differentiation of pluripotent stem cells offers exciting prospects for the generation of RGCs. By differentiating in suspension culture, Sasai’s group first demonstrated that hESCs can self-organised to form optic cup structures consisting of RPE cells and stratified neural retina with photoreceptors and RGCs^51^. This method has been modified and adapted by multiple groups^64^,^65^,^66^,^67^. In particular, a recent study successfully generated optic vesicle-like structures for modeling glaucoma in hiPSCs^67^. This differentiation strategy elegantly recapitulates the auto-regulation and patterning of tissues that occurs during optic cup development, allowing for study of RGCs *in vitro* and potentially the interaction of RGCs with other cell types in the neural retina. In comparison, our protocol generates enriched population of RGCs in adherent culture of RGCs that is easier to handle than suspension culture. In regards to modeling optic neuropathies, this study provides a complimentary approach to study RGCs specifically, compared to the optic cup differentiation approach to study multiple cell types in the neural retina. In summary, here we describe a protocol for differentiation and enrichment of RGCs using hESCs. Establishment of protocols for pluripotent stem cell-derived RGCs offer exciting potentials for modeling optic neuropathic diseases and developing RGC replacement therapies.

## Methods

### Ethics

All experimental work performed in this study was approved by the Human Research Ethics committees of the Royal Victorian Eye and Ear Hospital (11/1031H, 13/1151H-004) and the University of Melbourne (0605017) and carried out in accordance with the National Health & Medical Research Council of Australia (NHMRC) and conformed with the Declarations of Helsinki.

### Cell Culture

H9 hESCs were cultured on mitotically-inactivated mouse embryonic fibroblasts (MEFs) in DMEM-F12 with GlutaMAX, 20% knockout serum replacement (KSR), 10 ng/ml bFGF, 0.1 mM NEAA, 100 μM β-mercaptoethanol and 0.5 × penicillin/streptomycin (All from Invitrogen). hESCs were passaged as small cell clumps with accutase (Sigma) every seven days and kept in a 5% CO_2_ incubator.

### Retinal differentiation of hESCs

Undifferentiated H9 hESCs (WiCell) were maintained in a tissue-culture treated 6-well plate for seven days, followed by subsequent formation of embryoid bodies (day 0) in a 6-well ultra-low attachment plate (Corning). The embryoid bodies were cultured in RGC differentiation medium 1, consisting of DMEM F12 with GlutaMAX, 10% KSR, 1X B27 (Invitrogen), 1 ng/ml noggin (Sapphire Biosciences), 1 ng/ml DKK1 (Peprotech) and 5 ng/ml IGF1 (Peprotech). On day four, embryoid bodies were plated down into a 6-well plate pre-coated with poly-L-lysine (100 μg/well, Sigma) and Matrigel (40 μl/well, BD Biosciences). Plated embryoid bodies were further differentiated in RGC differentiation medium 2, consisting of DMEM F12 with GlutaMAX, 10% KSR, 1X B27, 1X N2 (Invitrogen), 10 ng/ml noggin, 10 ng/ml DKK1, 10 ng/ml IGF1 and 5 ng/ml bFGF. Medium was changed every two or three days.

### Enrichment of hESC-derived RGCs

Day-30 retinal differentiated hESCs were treated with accutase (Sigma-Aldrich) and dissociated into a single cell suspension by pipetting up and down and filtered using a 100 μm nylon strainer (Falcon) to eliminate large cell clumps. MACS isolation was performed to enrich for THY1.1 positive RGCs using a MACS manual separator or the MultiMACS Separator, following the manufacturer’s instructions (Miltenyi Biotech). Briefly, dissociated cells were washed with PBS and labelled with THY1.1 microbeads (Miltenyi Biotech) for 15 minutes at 4 °C. The cell samples were then washed with PBS and isolated using a LS MACS column. THY1.1 enriched cells were collected and plated down onto glass coverslips pre-coated with poly-L-lysine and Matrigel in 24-well or 6-well plates. THY1.1 enriched cells were grown in the presence of RGC differentiation medium 2 for a further 15 days prior to analysis.

### Quantification of hESC-derived RGCs

On day 30 of retinal differentiation, quantification of THY1.1 positive cells was performed by counting the number of cells isolated by THY1.1 MACS (THY1.1 positive cells), divided by the number of total cells prior to MACS isolation. Cell number was determined using the Countess Automated Cell Counter (Invitrogen). For quantification of RGC enrichment post-MACS, the THY1.1 positive or negative population was immunostained using standard procedures^58^, using primary antibodies against BRN3A (#MAB1585, Millipore) or THY1.1 (#MAB1406, Millipore), followed by the corresponding AlexaFluor 488. Flow cytometry analysis of the samples was performed using a MACSquant Analyser (Miltenyi Biotech).

### Quantitative PCR

RNA samples were extracted using the RNeasy kit and treated with DNase 1 to eliminate DNA contamination (All from Qiagen). cDNA synthesis was performed using the High capacity cDNA reverse transcription kit (ABI). Taqman assay was performed using the Taqman fast advanced master mix (ABI) in triplicates following manufacturer’s protocol, using probes for *RAX* (Hs00429459_m1), *MITF* (Hs01117294_m1), *ATOH7* (Hs00376955_s1), *PAX6* (Hs00240871_m1), *BRN3B* (Hs00231820_m1), *ISL1* (Hs00158126_m1), *OPN4* (Hs00264482_m1) and the housekeeping genes *β-ACTIN* (Hs99999903_m1) or *18S* (Hs99999901_s1). Samples were processed using the Step One plus real time PCR system (ABI). The Ct threshold was set using the parameters by the Stepone software (ABI) and checked manually. The results were normalised to the housekeeping genes β-actin or 18S and analysed using the ΔΔCt method. Results were presented in log2 fold changes compared to undifferentiated hESCs and statistical tests were performed using one-way ANOVA test with Dunnett post-hoc test.

### Immunocytochemistry

hESC-derived RGCs were immunostained following standard procedures^68^,^69^, using primary antibodies against βIII-TUBULIN (#MAB1637, Millipore), HUC/D (#A21271, Invitrogen), BRN3A (#MAB1585, Millipore), NEFM (#AB5735, Millipore), RPE65 (#AB78036, Abcam) and CRALBP (#AB15051, Abcam). The samples were then stained with the corresponding AlexaFluor 488, 568 or 594 secondary antibodies, followed by counterstain with DAPI (Invitrogen) and visualized using a fluorescent microscope (Nikon TE2000 or Zeiss Axio Imager M2). Negative isotype controls (Dako) showing absence of staining were used to confirm the specificity of the primary antibodies.

### Whole transcriptome analysis by RNAseq

Total RNA were extracted using the RNeasy kit and treated with DNase 1 (Qiagen). Triplicate sets of RNA samples were obtained from undifferentiated H9 and Day 45 hESC-RGCs. Whole transcriptome libraries generation and rRNA depletion were performed by the Australian Genomic Research Facility. Pair-end sequencing (100 bp) was performed using an Illumina HiSeq 2000. Bioinformatic analysis was performed using ExAtlas as described previously^70^. Transcriptome data of human RGC layer and outer retina layers were obtained from Kim *et al*.^33^, data of all other human tissues are obtained from NCBI Gene Expression Omnibus database (GSE15175, GSE21668, GSE26862, GSE29783, GSE54186, GSE3526). Expression data for H9 and hESC-RGCs in this study are also available (GSE84639).

### Electrophysiology

Functional analysis by electrophysiology was performed in retinal neurons and RGCs derived from hESCs as described^71^. Briefly, whole-cell patch-clamp recordings were measured in individual cultured neurons, using borosilicate microelectrodes (2–6 MOhm) filled with internal solution containing: 115 mM K-gluconate, 10 mM HEPES, 7 mM KCl, 5 mM EGTA, 2 mM Na_2_ATP, 2 mM MgATP, 0.5 mM Na_2_GTP (pH 7.3; 290–295 mOsmol/kg). Signals were recorded using a MultiClamp 700B amplifier and data acquisition system (Digidata 1440A) and AxoGraph X analysis software.

### Quantification of axonal transport kinetics of mitochondria

Enriched hESC-RGCs were stained with Mitotracker Red CMXRos (100 nM, Invitrogen) and subsequently imaged using a Nikon C1 confocal mounted on a Ti-E inverted microscope time-lapse analysis. Images were recorded every 20 seconds for up to 15 minutes. To analyse axonal transport, over 1000 mitochondria clusters were tracked to determine the average speed, maximum speed, speed variance and instantaneous acceleration of axonal transport using Imaris v7.6.5 software.

## Additional Information

**How to cite this article**: Gill, K. P. *et al*. Enriched retinal ganglion cells derived from human embryonic stem cells. *Sci. Rep.* **6**, 30552; doi: 10.1038/srep30552 (2016).

## Supplementary Material

Supplementary Information
