# Supplementary material for: Enriched retinal ganglion cells derived from human embryonic stem cells
Source: Sci Rep. 2016 Aug 10;6:30552. doi: 10.1038/srep30552 (PMC4978994; doi:10.1038/srep30552)
Supplement: Supplementary Information [file srep30552-s1.pdf]

Supplementary Information:

Gill KP, Hung SSC, Sharov A, Lo CY, Needham K, Lidgerwood GE, Jackson S, Crombie DE, Nayagam B, Cook AL, Hewitt AW, Pébay A, Wong RCB (2016) Enriched retinal ganglion cells derived from human embryonic stem cells.

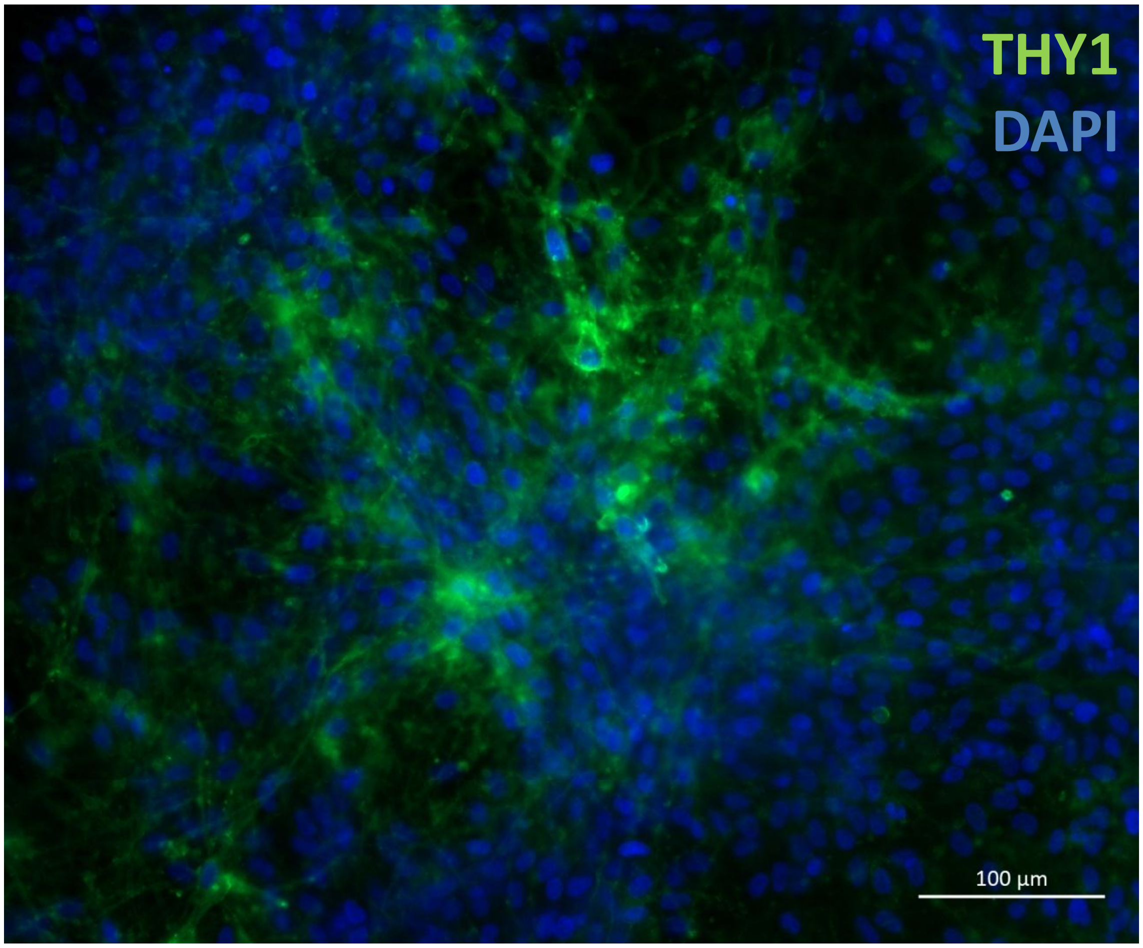

Supplementary figure 1: THY1 staining in hESC-derived RGCs.
Immunocytochemistry of THY1 (green) and DAPI in day 62 hESC-derived RGCs. Scale bar = 100 µm.

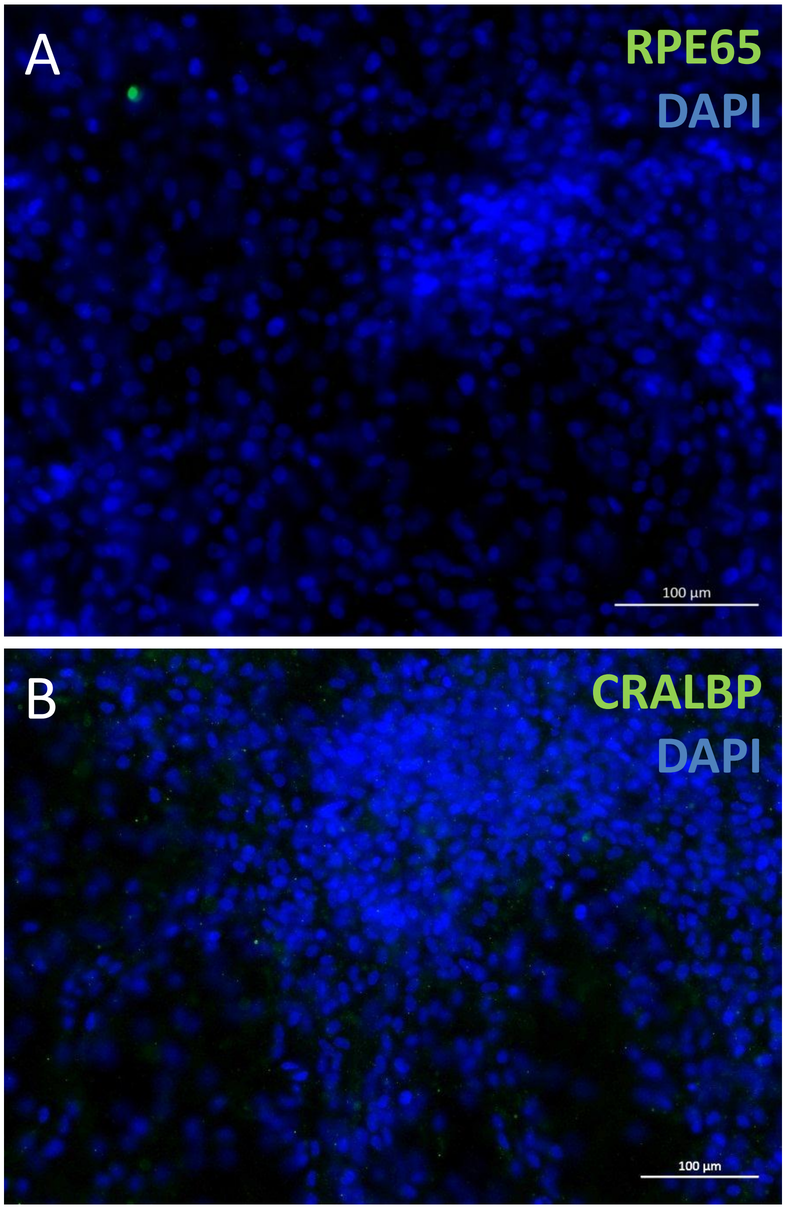

Supplementary figure 2: Absence of RPE cells and Müller cells in hESC-derived RGC culture. Immunocytochemistry of A) RPE65 as RPE marker; and B) CRALBP as Müller cell marker in day 62 hESC-derived RGC culture. Scale bars = 100 µm.
